# Risk Factors for Small‐for‐Size Syndrome Grade B/C After Simultaneous Splenectomy in Adult Living‐Donor Liver Transplantation

**DOI:** 10.1002/ags3.70181

**Published:** 2026-01-28

**Authors:** Kyohei Yugawa, Takeo Toshima, Shinji Itoh, Takashi Motomura, Shohei Yoshiya, Norifumi Iseda, Tomoharu Yoshizumi

**Affiliations:** ^1^ Department of Surgery and Science, Graduate School of Medical Sciences Kyushu University Fukuoka Japan

**Keywords:** living‐donor liver transplantation, prognosis, small‐for‐size syndrome, splenectomy

## Abstract

**Background:**

Small‐for‐size syndrome (SFSS) after adult living‐donor liver transplantation (LDLT) is a poor prognostic condition. Splenectomy (Spx) has recently been reported to help prevent SFSS and improve outcomes. This study aimed to identify risk factors for SFSS that occur despite Spx at the time of LDLT.

**Methods:**

This single‐center retrospective study included 973 LDLT recipients from 2001 to 2024. Of these, 577 who underwent concomitant Spx were analyzed. SFSS grading followed the 2023 International Liver Transplantation Society consensus criteria. Prognostic factors associated with SFSS grade B/C, which required perioperative portal vein inflow control, were identified.

**Results:**

SFSS grade B/C occurred in 105 patients (18.2%) and was associated with significantly worse prognosis than grade 0/A (1‐year survival: 93.9% vs. 81.9%, *p* < 0.0001). The grade B/C group had higher levels of Model for End‐stage Liver Disease (MELD) score (median 18 vs. 15, *p* < 0.0001), neutrophil‐to‐lymphocyte ratio (NLR) (3.9 vs. 2.7, *p* < 0.0001), donor age (37 vs. 40 years, *p* = 0.0370), and donor body mass index (BMI) (22.7 vs. 21.8 kg/m^2^, *p* = 0.0093). Multivariate logistic regression identified NLR of ≥ 4.5, MELD score of ≥ 30, and donor age of ≥ 50 years, as independent predictors.

**Conclusions:**

SFSS grade B/C still occurred in nearly one in five patients who underwent Spx for SFSS risk mitigation during LDLT. Patients with a high recipient NLR, MELD score, or donor age are at increased risk of SFSS despite Spx. Future strategies should emphasize careful donor selection and perioperative portal inflow modulation for high‐risk cases.

## Graphical abstract

In a single‐center cohort of 577 adult LDLT recipients who underwent simultaneous splenectomy, clinically significant SFSS grade B/C (ILTS‐iLDLT‐LTSI 2023) occurred in 18.2% and was associated with inferior graft survival. Multivariate analysis identified MELD ≥ 30, NLR ≥ 4.5, and donor age ≥ 50 years as independent risk factors, which risk rising cumulatively as these factors coexisted. These findings support stricter donor selection (younger, higher quality grafts) and careful preparation for portal inflow modulation in high‐risk recipients.

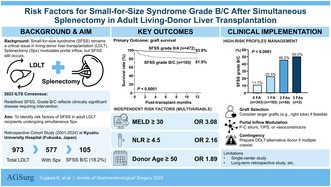

**Abbreviations**
BMI: body mass index
CI: confidence interval
DDLT: deceased‐donor liver transplantation
GV/SLV: graft volume‐to‐standard liver volume
ICU: intensive care unit
ILTS‐iLDLT‐LTSI: International Liver Transplantation Society–International Living Donor Liver Transplantation Group–Liver Transplant Society of India
LDLT: living‐donor liver transplantation
MELD: model for end‐stage liver disease
NLR: neutrophil‐to‐lymphocyte ratio
OR: odds ratio
POD: postoperative day
PT‐INR: prothrombin time–international normalized ratio
SFSS: small‐for‐size syndrome
Spx: splenectomy

## 1 Introduction

Living‐donor liver transplantation (LDLT) offers superior overall survival compared with deceased‐donor liver transplantation (DDLT) because LDLT recipients are typically transplanted with less advanced liver disease, spend less time on the waiting list, and face a lower risk of rejection. The use of LDLT continues to expand for patients with end‐stage liver disease who have access to a suitable donor [1, 2]. However, graft size remains a critical issue in LDLT because it often involves grafts that are just large enough to meet the minimum size requirement [3]. Smaller grafts may be unable to fulfill all the metabolic, synthetic, and hemodynamic demands of the recipient, potentially leading to early allograft dysfunction [4].

In addition, a post‐LDLT case of graft insufficiency—characterized by delayed cholestasis, prolonged ascites output, stable vascular flow, and normalized transaminase levels—was reported and termed small‐for‐size syndrome (SFSS) [5]. This complication can be catastrophic and should therefore be anticipated or avoided through appropriate preoperative planning. In a consensus report by the International Liver Transplantation Society–International Living Donor Liver Transplantation Group–Liver Transplant Society of India (ILTS‐iLDLT‐LTSI), new grading criteria and definitions for SFSS were proposed to improve understanding of its pathophysiology and to guide the adoption of optimal preventive strategies.

Splenectomy (Spx) is performed as a form of portal inflow modulation during LDLT and has been reported to increase graft survival by approximately 10% [3], as splenic flow can account for up to half of the total portal flow [6]. Excessive portal inflow and elevated portal venous pressure negatively impact graft function, contributing to SFSS or early allograft dysfunction after LDLT [7]. Spx is, therefore, a theoretically reasonable method for modulating an abnormal portal circulatory system [8]. However, it is also true that some patients do not survive even after LDLT with simultaneous Spx. It is known that most of the patients who experience fatal outcomes develop SFSS [9], suggesting that it is necessary to assess the relationship between SFSS and Spx. As such, the impact of this strategy should be reevaluated in light of the new SFSS criteria [10]. Identifying predictors of SFSS even after Spx may aid in the management of patients who have undergone LDLT or are awaiting DDLT. This study aimed to identify predictive factors for SFSS following simultaneous Spx during LDLT to support decision‐making regarding indications and graft selection.

## 2 Methods

### 2.1 Patients and Ethics

This study included adult transplant recipients who underwent LDLT at Kyushu University Hospital (Fukuoka, Japan) between 2001 and 2024. Anonymized preoperative and postoperative clinical data were obtained from both electronic and paper records. The study protocol was approved by the Ethics Committee of the Kyushu University Hospital Institutional Review Board (approval number: 2019‐186) and conducted in accordance with the Code of Ethics of the World Medical Association (Declaration of Helsinki). All patients provided consent for the use of their clinical data through an opt‐out process.

### 2.2 Indications for Simultaneous Spx and LDLT

The indications for simultaneous Spx during LDLT have been previously described [9]. At our institution, the following three criteria have been established for performing Spx: a low actual graft weight‐to‐standard liver weight (GW/SLW) ratio (≤ 35%); the presence of portal hypertension—defined by a large portosystemic shunt (≥ 10 mm) [11], splenomegaly, or high‐risk esophagogastric varices; and high portal pressure (> 20 mmHg) after graft implantation. All recipients who underwent Spx during LDLT received preoperative vaccinations regardless of etiology, based on evidence of increased risk of severe sepsis after Spx.

### 2.3 Surgical Procedures

The graft procurement technique and transplant recipient surgery have been previously described [12]. Spx was performed using a vessel sealing system (Ligasure; Covidien Japan, Tokyo, Japan) and an automatic suturing device (Endo GIA; Covidien Japan, Tokyo, Japan, or powered ECHELON; ETHICON, New Brunswick, NJ, USA), as previously reported [3]. Because of severe adhesions around the spleen, simultaneous Spx was not performed in patients who had undergone partial splenic embolization prior to LDLT. Regarding portosystemic shunt closure, we aimed to ligate any existing shunts ≥ 10 mm in diameter [11] because the presence of a large splenorenal shunt serves as an indirect indicator of potentially severe portal hypertension.

### 2.4 Graft Selection

Graft selection and its indications have been previously described [9]. Donors were required to be either spouses or within the third degree of consanguinity with the recipients and between 20 and 65 years of age [13]. Preoperative volumetric analysis and delineation of vascular anatomy were performed using three‐dimensional computed tomography. Graft volume (GV) was predicted using computed tomography volumetric analysis. The type of graft was selected based on the preoperatively predicted GV‐to–standard liver volume (SLV) ratio. GV/SLV was calculated as GV (mL)/{706.2 × body surface area (m^2^) + 2.4} (mL) [14]. A left lobe (LL) graft, with or without the caudate lobe, was used if the estimated GV/SLV was ≥ 35%. A right lobe (RL) graft was selected if the GV/SLV of the LL (with caudate lobe) was < 35% and the donor's predicted remnant liver volume was ≥ 35%. A right posterior sector graft was considered when the remnant liver volume after right hepatectomy was < 35% [15]. When using a RL graft, the middle hepatic vein tributaries draining segments 5 and 8 and/or the inferior right hepatic vein were evaluated. Reconstruction of these veins was performed if their diameter was ≥ 5 mm or if their volume was ≥ 10% of the GV [16]. Graft selection was individualized, integrating anatomical variations, donor age, anticipated graft quality, and the severity of the recipient's overall clinical status. In all cases, donor safety constituted the overarching priority. Actual graft weight (GW)–to–standard liver weight (SLW) was calculated as GW (g)/{706.2 × body surface area (m^2^) + 2.4} (g), with the conversion ratio between liver weight (g) and volume (mL) set to 1.0.

### 2.5 Postoperative Management

Recipients' perioperative management, including immunosuppression regimens, has been described previously [9, 17]. Immunosuppression was initiated using a protocol based on tacrolimus or cyclosporine A, in combination with a steroid and/or mycophenolate mofetil. All transplant recipients underwent monthly follow‐up evaluations. The median follow‐up period was 2239 days (interquartile range, 729–4050 days). Vaccination against *Streptococcus pneumoniae* was administered at least 1 year after LDLT. Transplant recipients who underwent simultaneous Spx and LDLT were advised to receive a booster vaccination every 5 years. Throughout the observation period, no adverse effects related to the vaccine were recorded among patients who underwent simultaneous Spx.

### 2.6 Definition of SFSS

SFSS is defined as prolonged functional cholestasis and intractable ascites. SFSS was graded according to the criteria proposed by the ILTS‐iLDLT LTSI Consensus Conference working group [18], as follows:

Grade A (pre‐SFSS): total bilirubin of > 5 mg/dL on postoperative day (POD) 7 and either total bilirubin of > 5 mg/dL or ascites output of 1 L/day on POD14.

Grade B (portal hypertensive phase): total bilirubin of > 10 mg/dL or prothrombin time–international normalized ratio (PT‐INR) of > 1.6 on POD7, and total bilirubin of > 10 mg/dL with ascites output of 1 L/day on POD14.

Grade C (liver failure phase): total bilirubin of > 20 mg/dL and PT‐INR of > 1.6 on POD14.

In this study, SFSS grades B and C were considered clinically significant outcomes.

### 2.7 Statistical Analysis

Data are presented as mean, median, frequency, and percentage. Continuous variables were compared using the Mann–Whitney *U* test or the Kruskal–Wallis test and are reported as median and interquartile range. Categorical variables were compared using the *χ* ^2^ test or Fisher's exact test. The receiver operating characteristic (ROC) curves with the Youden's index correction were estimated to determine the optimal cutoff values for analyzing the risk of SFSS grade B/C after LDLT simultaneous Spx. Univariate and multivariate analyses were performed using logistic regression. All statistical tests were two‐sided, with a *p* value of < 0.05 considered statistically significant. Analyses were conducted using JMP 18 Pro software (SAS Institute, Cary, NC, USA).

## 3 Results

### 3.1 Patients' Characteristics According to Presence of SFSS Grade B/C After LDLT With Simultaneous Spx

Of the 973 patients, 577 underwent simultaneous Spx and LDLT. In this study, SFSS grades B and C were considered clinically significant outcomes, hence all 577 patients were classified into two groups: SFSS grades 0/A and grades B/C. Table 1 compares the preoperative characteristics of patients with SFSS grades 0/A (*n* = 472, 81.8%) versus B/C (*n* = 105, 18.2%) after LDLT with simultaneous Spx. Compared with the SFSS 0/A group, patients in the SFSS B/C group had a greater proportion with preoperative intensive care unit (ICU) stays (*p* < 0.0001) and had a chronic kidney disease (CKD, *p* = 0.0034). They also had higher total bilirubin levels (*p* < 0.0001), a higher PT‐INR (*p* = 0.0073), and higher Model for End‐stage Liver Disease (MELD) scores (*p* < 0.0001). In addition, the neutrophil‐to‐lymphocyte ratio (NLR) was higher in the SFSS B/C group than in the SFSS 0/A group (median 3.9 vs. 2.7, *p* < 0.0001, Figure S1) and the lymphocyte‐to‐monocyte ratio was lower in the SFSS B/C group than in the SFSS 0/A group (*p* = 0.0012). The SFSS B/C group also more frequently received grafts from donors with an older aged and a higher body mass index (BMI) (*p* = 0.0370 and *p* = 0.0093, respectively). Table 2 presents the operative and postoperative characteristics of the two groups. Compared with the SFSS 0/A group, the SFSS B/C group had lighter grafts (*p* = 0.0063), lower actual GW/SLW ratio (*p* = 0.0298), lower graft‐to‐recipient weight ratio (*p* = 0.0372), and greater intraoperative blood loss (*p* < 0.0001). Although portal venous pressure (PVP) at closure was higher in the SFSS B/C group (*p* = 0.0375), no significant differences in PVP before or after Spx were observed between the two groups. Furthermore, there was significant difference between the groups in the post‐LDLT complications (Clavien‐Dindo grade ≥ IIIa, *p* < 0.0001).

**TABLE 1:** Baseline characteristics of patients according to the presence of SFSS grade B/C after LDLT simultaneous Spx.

Preoperative characteristics	SFSS grade 0/A (*n* = 472)	SFSS grade B/C (*n* = 105)	*p*
**Baseline recipient preoperative characteristics**			
Age, years	57 (50–63)	57 (49–64)	0.9623
Sex, F/M	253/219	63/42	0.2335
BMI, kg/m^2^	24.0 (21.5–26.3)	23.5 (21.7–25.8)	0.7243
Primary diagnosis, %			
Hepatocellular disease	353 (74.8%)	69 (65.7%)	0.2154
Acute liver failure	17 (3.6%)	6 (5.7%)	
Cholestatic disease	86 (18.2%)	27 (25.7%)	
Others	16 (3.4%)	3 (2.9%)	
Hepatocellular carcinoma, %	187 (39.6%)	33 (31.4%)	0.1642
NLR	2.70 (1.59–4.32)	3.94 (2.48–7.91)	< 0.0001**
LMR	2.82 (1.91–4.08)	2.12 (1.22–3.57)	0.0012*
ICU stay	21 (4.5%)	21 (20.0%)	< 0.0001**
Child‐Pugh grade C, %	350 (74.2%)	87 (82.9%)	0.0598
MELD score	15 (12–19)	18 (14–25)	< 0.0001**
Total bilirubin, mg/dL	3.3 (2.1–6.0)	7.0 (3.6–17.6)	< 0.0001**
Albumin, g/dL	2.7 (2.4–3.0)	2.6 (2.3–3.0)	0.2544
PT‐INR	1.45 (1.29–1.67)	1.53 (1.35–2.04)	0.0073*
Diabetes mellitus, %	92 (19.5%)	21 (20.0%)	0.9055
Chronic kidney disease, %	110 (23.3%)	39 (37.1%)	0.0034*
**Baseline donor characteristics**			
Age, years	37 (30–46)	40 (32–51)	0.0370*
Sex, F/M	191/281	45/60	0.6522
BMI, kg/m^2^	21.8 (20.3–23.6)	22.7 (21.0–24.3)	0.0093*
ABO, identical/compatible/incompatible	272/105/95	58/24/23	0.8901

*Note:* Data are presented as *N* or median (interquartile range, IQR).

Abbreviations: BMI, body mass index; ICU, intensive care unit; LDLT, living‐donor liver transplantation; LMR, lymphocyte‐to‐monocyte ratio; MELD, model for end‐stage liver disease; NLR, neutrophil‐to‐lymphocyte ratio; PT‐INR, prothrombin time–international normalized ratio; SFSS, small‐for‐size syndrome; Spx, splenectomy.

*p < 0.05, **p < 0.001.

**TABLE 2:** Operative and postoperative characteristics of patients according to the presence of SFSS grade B/C after LDLT simultaneous Spx.

	SFSS grade 0/A (*n* = 472)	SFSS grade B/C (*n* = 105)	*p*
**Operative characteristics**			
Graft weight, g	482 (400–575)	448 (356–528)	0.0063*
Spleen weight, g	336 (221–514)	347 (200–467)	0.4822
Graft‐to‐spleen weight ratio	1.41 (0.91–2.14)	1.34 (0.86–2.15)	0.6895
Actual GW/SLW, %	40.5 (35.5–46.8)	38.1 (32.7–46.5)	0.0298*
GRWR, %	0.76 (0.66–0.89)	0.72 (0.63–0.87)	0.0372*
Graft type, right, %	240 (55.2%)	49 (48.5%)	0.2266
Warm ischemic time, min	40 (34–49)	39 (33–49)	0.5348
Cold ischemic time, min	101 (69–151)	94 (70–150)	0.7577
Duration of surgery, min	713 (634–803)	739 (645–878)	0.0606
Intraoperative blood loss, mL	3655 (2012–6348)	5840 (3236–10 939)	< 0.0001**
Shunt, %	229 (48.5%)	50 (47.6%)	0.8677
Portal venous pressure at open, mmHg	25 (22–29)	25 (21–29)	0.5413
Portal venous pressure after reperfusion, mmHg	18 (16–22)	19 (16–22)	0.2169
Portal venous pressure at closure, mmHg	15 (13–17)	16 (14–18)	0.0375*
Portal venous pressure change after Spx, %	−17.4 (−29.6 to −5.0)	−15.8 (−28.6 to −4.8)	0.7502
Portal venous flow after reperfusion, mL/s	1460 (1120–1960)	1380 (1120–1960)	0.5327
**Postoperative characteristics**			
Day 7 PT‐INR	1.11 (1.03–1.20)	1.18 (1.10–1.28)	< 0.0001**
Day 7 total bilirubin, mg/dL	4.0 (2.6–5.9)	12.2 (10.4–14.2)	< 0.0001**
Day 14 PT‐INR	1.07 (1.01–1.15)	1.14 (1.05–1.23)	< 0.0001**
Day 14 total bilirubin, mg/dL	2.1 (1.3–4.0)	11.2 (7.2–17.3)	< 0.0001**
Day 14 ascites, mL	0 (0–320)	495 (88–1024)	< 0.0001**
Post‐LDLT complications (Clavien‐Dindo ≥ IIIa, %)	73 (15.5%)	37 (35.2%)	< 0.0001**

*Note:* Data are presented as *N* or median (IQR).

Abbreviations: GRWR, graft recipient weight ratio; GW/SLW, graft weight‐to‐standard liver weight; LDLT, living‐donor liver transplantation; PT‐INR, prothrombin time–international normalized ratio; SFSS, small‐for‐size syndrome; Spx, splenectomy.

*p < 0.05, **p < 0.001.

### 3.2 LDLT Outcomes

**FIGURE 1:**
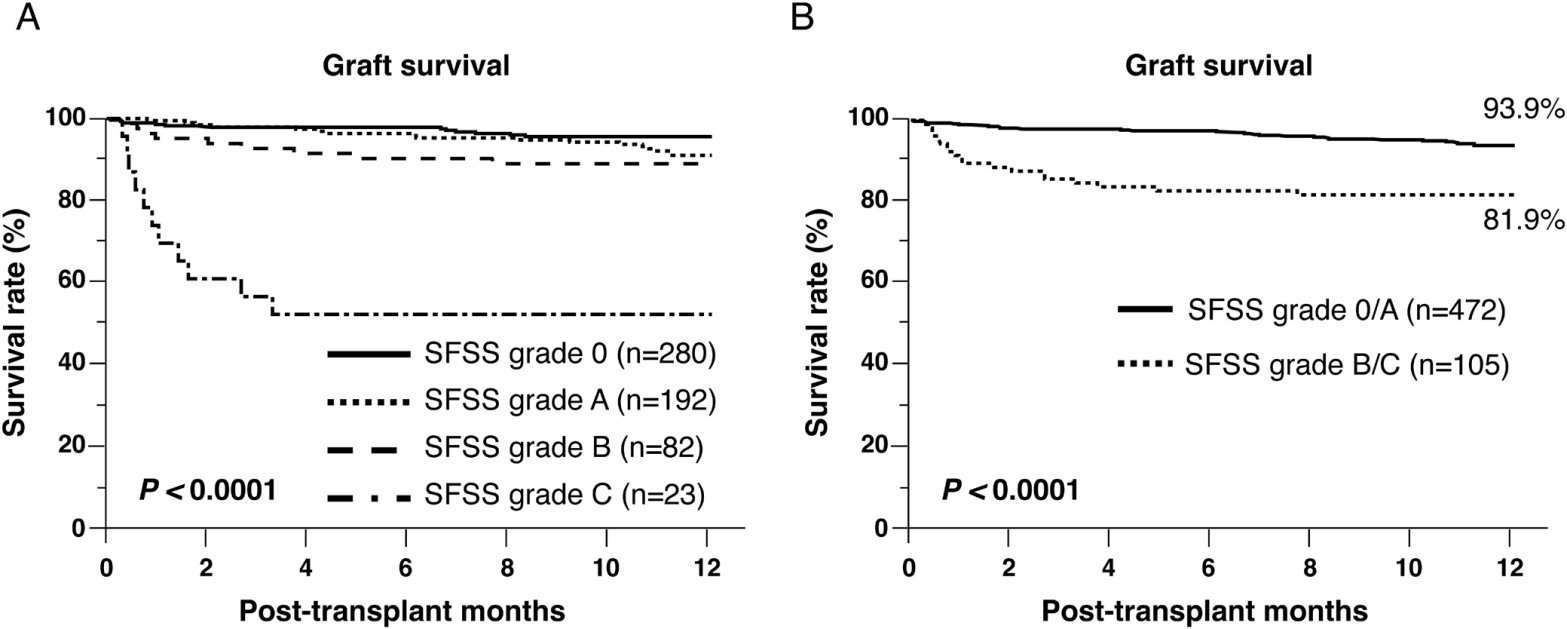
Comparison of graft survival according to the SFSS grading system: (A) standard SFSS grading and (B) SFSS grade 0/A versus B/C groups in patients who underwent simultaneous Spx during LDLT. LDLT, living‐donor liver transplantation; SFSS, small‐for‐size syndrome; Spx, splenectomy.

Kaplan–Meier curves comparing by standard SFSS grades showed that graft survival after LDLT with Spx was significantly shorter in the SFSS grade C compared to the other grades (log‐rank test, *p* < 0.0001, Figure 1A). Next, survival curves comparing patients with SFSS 0/A and B/C showed that graft survival after LDLT was significantly shorter in the SFSS B/C group than in the SFSS 0/A group following simultaneous Spx (log‐rank test, *p* < 0.0001, Figure 1B).

### 3.3 Independent Predictors of SFSS in Patients Who Underwent Simultaneous Spx During LDLT

An NLR of 4.5 (area under ROC curve, AUC = 0.650, sensitivity 47.6% and specificity 77.5%) and LMR of 2.2 (AUC = 0.601, sensitivity 51.9% and specificity 68.3%) were the best cutoff values for the SFSS grade B/C. The cutoff values of MELD score and donor age were determined by previous reports [19, 20]. Univariate analysis revealed that a higher MELD score, higher NLR, lower LMR, CKD, and older donor age were significant risks of SFSS grade B/C. Specifically, the presence of a MELD score ≥ 30 (odds ratio [OR], 5.43; 95% confidence interval [CI], 2.46–12.1; *p* < 0.0001), NLR of ≥ 4.5 (OR, 4.90; 95% CI, 1.86–4.51; *p* < 0.0001), LMR < 2.2 (OR, 2.22; 95% CI, 1.44–3.41; *p* = 0.0003), CKD (OR, 1.94; 95% CI, 1.23–3.904; *p* = 0.0044), and donor age ≥ 50 years (OR, 1.81; 95% CI, 1.09–2.95; *p* = 0.0224) were all significantly associated with SFSS grade B/C in patients who underwent LDLT with simultaneous Spx. In the multivariate analysis using these four factors, four remained significant predictors following: MELD score ≥ 30 (OR, 3.08; 95% CI, 1.27–7.45; *p* = 0.0130), NLR of ≥ 4.5 (OR, 2.16; 95% CI, 1.21–3.88; *p* = 0.0093), and donor age ≥ 50 years (OR, 1.89; 95% CI, 1.12–3.14; *p* = 0.0186) (Table 3). In addition, 267 cases with complete data in which Spx was not performed during LDLT were analyzed to identify risk factors for SFSS. Among these, SFSS was observed in 76 cases (28.5%). Based on a logistic regression model, multivariate analysis did not identify any independent risk factors (Table S1).

**TABLE 3:** Univariate and multivariate analyses for risk factors of SFSS grade B/C after LDLT simultaneous Spx (logistic regression analysis).

Variable	Univariate			Multivariate		
	OR	95% CI	*p*	OR	95% CI	*p*
Recipient sex: male	0.77	0.50–1.18	0.2319			
Recipient age ≥ 60 years	1.23	0.80–1.88	0.3503			
Liver cause: acute liver failure	2.04	0.77–4.91	0.1462			
MELD score ≥ 30	5.43	2.46–12.1	< 0.0001**	3.08	1.27–7.45	0.0130*
NLR ≥ 4.5	4.90	1.86–4.51	< 0.0001**	2.16	1.21–3.88	0.0093*
LMR < 2.2	2.22	1.44–3.41	0.0003**	1.09	0.61–1.94	0.7620
Chronic kidney disease	1.94	1.23–3.04	0.0044*	1.33	0.80–2.19	0.2671
Portosystemic shunt	0.96	0.63–1.47	0.8677			
Donor sex: male	0.91	0.59–1.40	0.6528			
Donor age ≥ 50 years	1.81	1.09–2.95	0.0224*	1.89	1.12–3.14	0.0186*
Donor BMI ≥ 25 kg/m^2^	1.12	0.56–2.08	0.7395			
Graft type: right lobe	0.77	0.50–1.18	0.2272			
GV/SLV < 35%	1.53	0.94–2.44	0.0843			
GRWR < 0.6	1.38	0.73–2.49	0.3059			
Portal venous pressure at closure ≥ 20 mmHg	1.92	0.81–4.18	0.1312			

Abbreviations: BMI, body mass index; CI, confidence interval; GRWR, graft recipient weight ratio; GV/SLV, graft volume‐to‐standard liver volume; ICU, intensive care unit; LDLT, living‐donor liver transplantation; MELD, model for end‐stage liver disease; NLR, neutrophil‐to‐lymphocyte ratio; OR, odds ratio; SFSS, small‐for‐size syndrome; Spx, splenectomy.

*p < 0.05, **p < 0.001.

**FIGURE 2:**
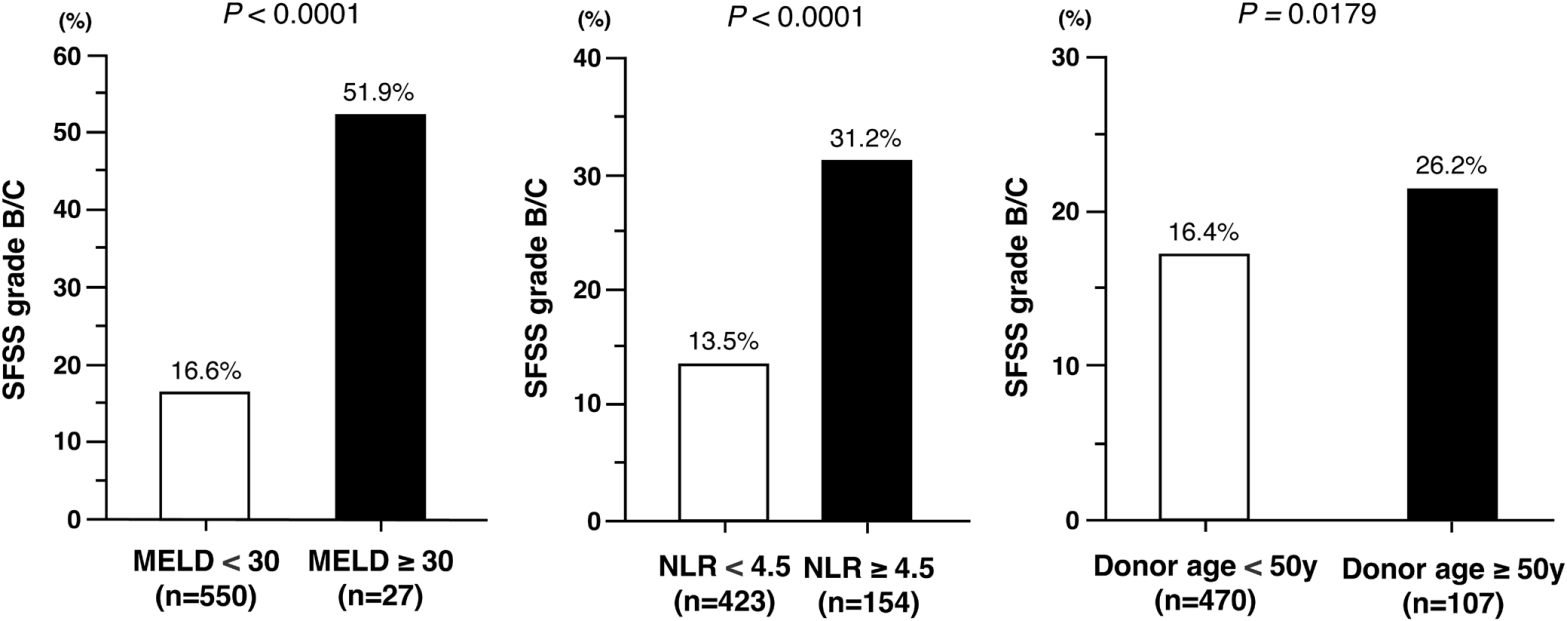
Incidence of SFSS grade B/C in patients with a MELD score of ≥ 30, an NLR of ≥ 4.5, and donor age ≥ 50 years who underwent simultaneous Spx during LDLT. LDLT, living‐donor liver transplantation; MELD, model for end‐stage liver disease; NLR, neutrophil‐to‐lymphocyte ratio; SFSS, small‐for‐size syndrome; Spx, splenectomy.

**FIGURE 3:**
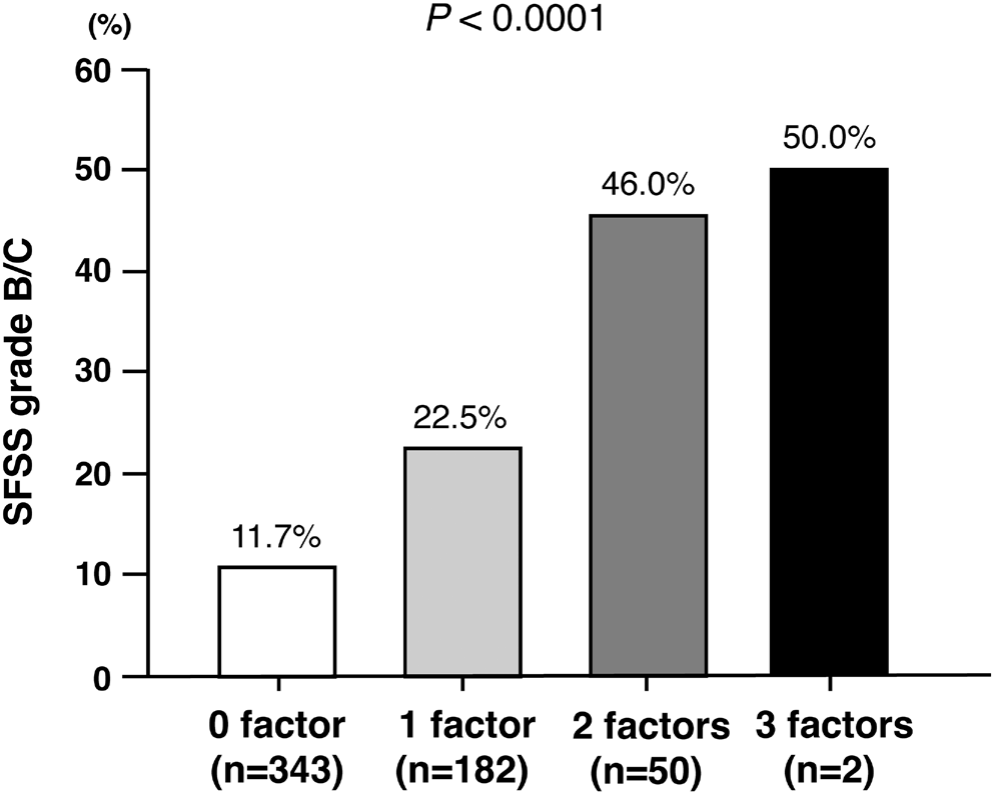
Frequency of SFSS grade B/C increased with the number of independent risk factors in patients who underwent simultaneous Spx during LDLT. LDLT, living‐donor liver transplantation; SFSS, small‐for‐size syndrome; Spx, splenectomy.

The incidence rates of SFSS grade B/C for each factor are shown in Figure 2. SFSS grade B/C occurred at significantly higher rates in the MELD score ≥ 30 group (51.9%, *p* < 0.0001), NLR ≥ 4.5 group (31.2%, *p* < 0.0001), and donor age ≥ 50 years (26.2%, *p* = 0.0179) than in their respective counterparts. Furthermore, the frequency of SFSS grade B/C increased progressively with the number of predictors present (Figure 3).

## 4 Discussion

This study is the first to clarify the independent predictors of SFSS grade B/C, as defined by the new criteria, following simultaneous Spx during LDLT using a relatively large study population. Spx is recommended as an effective and safe portal inflow modulation procedure, with a positive impact on postoperative complications and prognosis in patients undergoing LDLT. However, despite performing simultaneous Spx during LDLT, SFSS still occasionally occurred, and grade B/C was significantly associated with poorer graft survival compared with SFSS grade 0/A. Our findings indicate that pre‐LDLT patients with a MELD score of ≥ 30, with an NLR of ≥ 4.5, and who receive grafts from donors with an aged ≥ 50 years are at increased risk of developing SFSS grade B/C, even after simultaneous Spx during LDLT.

SFSS was first described by Ben‐Haim et al. [21] to characterize post‐LDLT grafts presenting with prolonged cholestasis and coagulopathy, despite normalization of transaminase levels. Understanding and analyzing the various perioperative factors that contribute to SFSS is essential for its prevention and management. An objective definition of SFSS was proposed by Soejima et al. [22] as a total bilirubin level of > 5 mg/dL and daily ascites output of > 1 L on POD14. Hill et al. [23] defined SFSS by the presence of significant cholestasis (serum bilirubin of > 10 mg/dL after POD7), coagulopathy (PT‐INR of > 1.5), and daily ascites output of > 2 L, in the absence of technical complications. Over time, the understanding of SFSS pathophysiology has advanced, leading to improved outcomes for at‐risk patients. However, there is still no consensus on the optimal strategies for SFSS prevention. The ILTS‐iLDLT‐LTSI Consensus Conference working group report introduced a new classification system for SFSS based on severity, offering medical, interventional radiology, and/or surgical guidelines for its management. From the framework, factors for predicting SFSS have been identified at each liver transplant center. However, as in our study, no other center has conducted a study using a new SFSS classification and in such a large cohort.

In LDLT, SFSS is more strongly influenced by lower graft quality, severe recipient status, and portal overflow when LL grafts are used rather than RL grafts because of the smaller number of hepatocytes in LL grafts. Accordingly, LL grafts may function as marginal grafts in selected contexts [15]. On the other hand, LL‐LDLT has achieved acceptable outcomes through selective use of younger donors and recipients with relatively lower MELD scores [24]. Therefore, consistent with a donor‐safety‐first policy, our institutional approach has prioritized LL‐LDLT when feasible; however, with LL grafts, targeted inflow control—such as Spx—has been emphasized to mitigate excessive portal inflow [15]. RL‐LDLT recipients are less likely to develop SFSS than LL‐LDLT recipients, whereas RL donors face higher rates of major complications and longer hospital stays [25]. For recipients with severe clinical status, RL grafts (accompanied by surgical portal inflow modulation) may therefore be preferable [26]. While RL use remains the most effective strategy for preventing SFSS, universal application is constrained by donor risk, underscoring the need to refine patient selection and identify recipient‐ and donor‐specific factors to individualize graft selection and inflow‐modulation strategies.

In addition to pre‐LDLT donor graft volume, the feasibility of Spx was evaluated based on monitoring PVP during LDLT. With regard to portosystemic shunts, large shunts were ligated to maintain adequate portal inflow and prevent the steal phenomenon, provided that PVP did not exceed 20 mmHg after test clamping. In multivariate models, classical hemodynamic demand surrogates (e.g., GV/SLV, GRWR, PVP) were not detected as independent risk factors. All patients in this cohort underwent simultaneous Spx and portosystemic shunt ligation when feasible, with a target intraoperative PVP < 20 mmHg. As shown in Table 2, PVP at open and after reperfusion did not differ between the two groups, and a difference was observed only at closure. This systematic inflow control likely normalized portal inflow regardless of graft size, thereby attenuating any independent effect of size. In this study, the multivariate model included MELD and NLR as measures of recipient demand and donor age as a proxy for graft quality, whereas classical size terms lost significance. These findings suggest that, in an Spx‐based inflow‐modulation strategy, “functional size” (recipient demand and graft quality) may be a stronger determinant of SFSS than “anatomical size.”

The NLR is a clinically accepted marker of systemic inflammation. In the LDLT setting, it was originally used as a surrogate for systemic inflammatory status and for predicting the development of sepsis in ICU patients. It has also been described as an independent predictor of decompensation in patients with cirrhotic liver disease [27]. Moreover, the NLR has been reported as a predictor of graft loss in patients who underwent LDLT with donors aged ≥ 50 years [17]. Consistent with these findings, the present study showed that a higher NLR is an independent risk factor for SFSS grade B or C after simultaneous Spx with LDLT. NLR has been reported to correlate with serum IL‐17 levels produced by Th17 cells, which recruit inflammatory cells to the liver and enhance inflammatory response in the microenvironment [28]. In recipients awaiting LDLT, a high NLR likely reflects increased IL‐17 levels and a highly inflamed environment that impairs liver regeneration—a key component in the pathogenesis of SFSS. However, no reports have elucidated the mechanism linking NLR and SFSS. Detailed mechanistic studies examining the interplay among NLR, Th17/IL‐17 signaling, and regenerative pathways in recipient are urgently required.

Currently, the following three major factors influencing successful LDLT are reported to be associated with graft failure after LDLT; recipient condition (as mentioned above), graft size, and graft quality [3]. Donor age is expected to increase with increased life expectancy of the general population, as such, there is need to carefully select graft in order to prevent graft failure. Donor age is correlated with graft quality, including liver steatosis or fibrosis through aging‐related genes [29]. This study population differed from our previous report [9] in that all recipients underwent LDLT with simultaneous Spx as a portal inflow modulation strategy, reflecting a high‐risk, more marginal LT setting. In such cases, graft quality is expected to play a more critical role in preventing SFSS. Accordingly, a more conservative donor‐age threshold may be clinically appropriate. In our cohort, only 18 donors were aged ≥ 60 years, raising concerns about low statistical power and unstable estimates at the cutoff; moreover, prior evidence supports the clinical relevance and feasibility of using a lower age boundary for graft selection [20]. From this perspective, setting the donor‐age cutoff at 50 years—rather than 60 years as in our previous cohort—may better align with donor selection for patients undergoing LDLT with Spx. The appropriateness of this donor‐age cutoff value has to be clarified in future studies using an independent validation cohort.

According to our results, recipients with factors such as a MELD score of ≥ 30 and/or an NLR of ≥ 4.5 are more likely to develop SFSS grade B/C, particular when the donor's age exceeds 50 years. In the pre‐LDLT setting, strict donor selection criteria should therefore be applied—preferably from donors aged under 50 years with minimal steatosis or fibrosis—and modifiable recipient factors should be optimized by treating active infections and improving nutritional status, with the aim of lowering the NLR when feasible. In the intra‐LDLT setting, careful preparation for portal inflow modulation is essential and venous outflow should be reconstructed aggressively (including V5/V8/IRHV for RL grafts and thorough MHV tributary management for LL grafts). Specifically, when these two or three risk factors are present, the probability of developing SFSS even after LDLT with simultaneous Spx reaches nearly 50%, resulting in a poor prognosis. In such high‐risk cases, it is considered desirable to perform liver transplantation after carefully considering sufficient strategies, such as planning for DDLT or living‐donor change. In LDLT recipients who have already undergone Spx, residual portal over‐inflow can be managed in several ways: a calibrated partial or temporary portocaval shunt to divert a controlled fraction of portal flow, a transjugular intrahepatic portosystemic shunt as a salvage option, and short courses of vasoconstrictors (including octreotide and vasopressin). However, these strategies are palliative, and re‐transplantation is required in severe cases of SFSS.

The main limitation is that this was a single‐center, long‐term retrospective study. Larger prospective studies, including external validation with independent cohorts, may be necessary to confirm the validity of these markers with higher sensitivity and specificity in patients undergoing LDLT. Despite this limitation, this is the first report with a relatively large sample size to demonstrate independent risk factors for SFSS in patients who underwent simultaneous Spx during LDLT.

In conclusion, this study suggests that patients undergoing simultaneous Spx during LDLT who have a MELD score of ≥ 30 and an NLR of ≥ 4.5, and receive grafts from donors with an aged ≥ 50 years may require substantially larger grafts—or, if possible, should be considered for a graft from a deceased donor.

## Author Contributions

**Kyohei Yugawa:** conceptualization, methodology, investigation, funding acquisition, writing – original draft, writing – review and editing, project administration, data curation. **Takeo Toshima:** supervision, data curation, investigation, funding acquisition, writing – review and editing. **Shinji Itoh:** writing – review and editing, supervision, data curation, methodology, investigation. **Takashi Motomura:** methodology. **Shohei Yoshiya:** methodology. **Norifumi Iseda:** methodology. **Tomoharu Yoshizumi:** conceptualization, investigation, funding acquisition, writing – review and editing, data curation, supervision, project administration, methodology.

## Funding

This study was supported by a JSPS KAKENHI, Grant‐in‐Aid from the Ministry of Health, Labour and Welfare, Japan (number JP‐25K12006 and 25K19738). The funding sources had no role in the collection, analysis, or interpretation of the data or in the decision to submit the article for publication.

## Ethics Statement

The authors have nothing to report.

## Consent

The authors have nothing to report.

## Conflicts of Interest

The authors declare no conflicts of interest, except that T.Y. serves as an associate editor of *Annals Gastroenterological Surgery*.

## Supporting information


**Figure S1:** Distribution map of NLR in the SFSS 0/A and B/C groups who underwent simultaneous Spx during LDLT. LDLT, living‐donor liver transplantation; NLR, neutrophil‐to‐lymphocyte ratio; SFSS, small‐for‐size syndrome; Spx, splenectomy.

**Table S1:** Multivariate analysis for risk factors of SFSS grade B/C after LDLT without Spx (*n* = 267, logistic regression analysis).
