# Supplementary figures and images for: Risk Factors for Small‐for‐Size Syndrome Grade B/C After Simultaneous Splenectomy in Adult Living‐Donor Liver Transplantation
Source: Ann Gastroenterol Surg. 2026 Jan 28;10(4):1165–73. doi: 10.1002/ags3.70181 (PMC13327040; doi:10.1002/ags3.70181)

Supplementary Figure 1

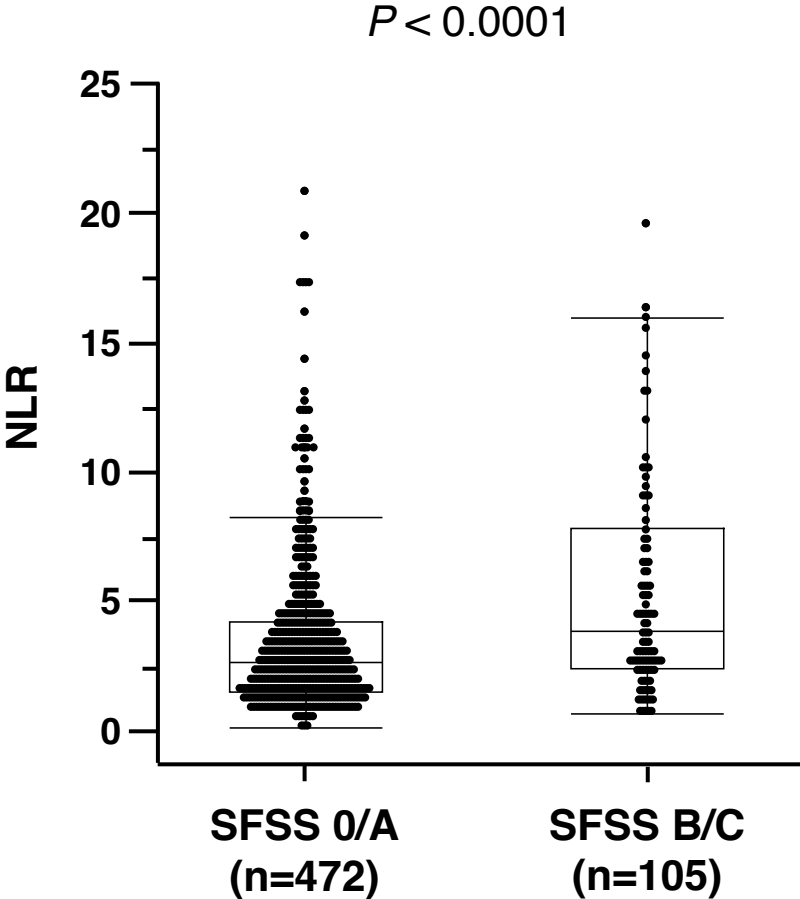

Supplement: Supplementary file 1 — Figure S1: Distribution map of NLR in the SFSS 0/A and B/C groups who underwent simultaneous Spx during LDLT. LDLT, living‐donor liver transplantation; NLR, neutrophil‐to‐lymphocyte ratio; SFSS, small‐for‐size syndrome; Spx, splenectomy. [file AGS3-10-1165-s001.pdf]
