# Supplementary material for: Risk Factors for Small‐for‐Size Syndrome Grade B/C After Simultaneous Splenectomy in Adult Living‐Donor Liver Transplantation
Source: Ann Gastroenterol Surg. 2026 Jan 28;10(4):1165–73. doi: 10.1002/ags3.70181 (PMC13327040; doi:10.1002/ags3.70181)
Supplement: Supplementary file 2 — Table S1: Multivariate analysis for risk factors of SFSS grade B/C after LDLT without Spx (n = 267, logistic regression analysis). [file AGS3-10-1165-s002.doc]

Supplementary Table 1. Multivariate analysis for risk factors of SFSS grade B/C after LDLT without Spx (n=267, logistic regression analysis).

|  |  | Univariate |  |
| --- | --- | --- | --- |
| Variable | OR | 95% CI | *P*-value |
| Recipient sex: male | 0.58 | 0.32-1.05 | 0.0697 |
| Recipient age ≥ 60 y | 0.92 | 0.48-1.78 | 0.8134 |
| Liver cause: acute liver failure | 0.62 | 0.28-1.38 | 0.2425 |
| MELD score ≥ 30 | 1.03 | 0.32-3.30 | 0.9628 |
| NLR ≥ 4.5 | 1.80 | 0.98-3.30 | 0.0582 |
| Portosystemic shunt | 1.39 | 0.67-2.89 | 0.3781 |
| Donor sex: male | 0.76 | 0.39-1.47 | 0.4172 |
| Donor age ≥ 50 y | 1.77 | 0.81-3.87 | 0.1548 |
| Donor BMI ≥ 25 kg/m^2^ | 1.28 | 0.58-2.82 | 0.5425 |
| Graft type: right lobe | 0.64 | 0.32-1.30 | 0.2181 |
| GV/SLV < 35% | 0.52 | 0.20-1.37 | 0.1856 |
| GRWR < 0.8 | 1.21 | 0.61-2.39 | 0.5885 |

BMI, body mass index; CI, confidence interval; GRWR, graft recipient weight ratio; GV/SLV, graft volume-to-standard liver volume; LDLT, living-donor liver transplantation; ICU, intensive care unit; MELD, model for end-stage liver disease; NLR, neutrophil-to-lymphocyte ratio; OR, odds ratio; SFSS, small-for-size syndrome; Spx, splenectomy, **P*<0.05, ***P*<0.001
